# Bayesian LASSO, Scale Space and Decision Making in Association Genetics

**DOI:** 10.1371/journal.pone.0120017

**Published:** 2015-04-09

**Authors:** Leena Pasanen, Lasse Holmström, Mikko J. Sillanpää

**Affiliations:** 1 Department of Mathematical Sciences, University of Oulu, Oulu, Finland; 2 Department of Biology, University of Oulu, Oulu, Finland; 3 Biocenter Oulu, Oulu, Finland; University of Miami, UNITED STATES

## Abstract

**Background:**

LASSO is a penalized regression method that facilitates model fitting in situations where there are as many, or even more explanatory variables than observations, and only a few variables are relevant in explaining the data. We focus on the Bayesian version of LASSO and consider four problems that need special attention: (i) controlling false positives, (ii) multiple comparisons, (iii) collinearity among explanatory variables, and (iv) the choice of the tuning parameter that controls the amount of shrinkage and the sparsity of the estimates. The particular application considered is association genetics, where LASSO regression can be used to find links between chromosome locations and phenotypic traits in a biological organism. However, the proposed techniques are relevant also in other contexts where LASSO is used for variable selection.

**Results:**

We separate the true associations from false positives using the posterior distribution of the effects (regression coefficients) provided by Bayesian LASSO. We propose to solve the multiple comparisons problem by using simultaneous inference based on the joint posterior distribution of the effects. Bayesian LASSO also tends to distribute an effect among collinear variables, making detection of an association difficult. We propose to solve this problem by considering not only individual effects but also their functionals (i.e. sums and differences). Finally, whereas in Bayesian LASSO the tuning parameter is often regarded as a random variable, we adopt a scale space view and consider a whole range of fixed tuning parameters, instead. The effect estimates and the associated inference are considered for all tuning parameters in the selected range and the results are visualized with color maps that provide useful insights into data and the association problem considered. The methods are illustrated using two sets of artificial data and one real data set, all representing typical settings in association genetics.

## Introduction

A large number of markers, segments of the DNA molecule, are commonly available in genetic studies involving association mapping and genomic prediction. Mapping studies focus on finding a few major genes called quantitative trait loci (QTL) out of a large number of markers. In genomic prediction, the number of markers included in the model depends on the genetic architecture and the extent of collinearity between markers. In these studies, all markers are included in the model *a priori* and variable selection is applied to arrive at a “sparse” subset of trait-associated marker effects *a posteriori*. Variable selection regularizes the problem so that the number of estimated non-zero marker effects hopefully becomes smaller than the number of observations leading to meaningful but downwardly biased effect estimates.

Bayesian variable selection methods have been widely applied for QTL mapping and genomic prediction [1]. Variable selection in these models typically uses either shrinkage analysis or auxiliary indicator variables (i.e., slab-and-spike variable selection). The former method specifies a marker effect prior that shrinks the effects of negligible markers heavily towards zero, while assigning large effects to important markers. In the latter technique, each marker has its own auxiliary indicator variable that has a much smaller prior probability for marker inclusion than marker exclusion. While Bayesian shrinkage models are common tools for genomic prediction, rigorous decision making in QTL mapping studies is still an open research problem with such models [2]. The methods developed in this paper provide novel tools for finding relevant genomic markers using the Bayesian LASSO [3, 4], a shrinkage-based method that uses a Laplacian prior distribution for marker effects. We consider four problems that need special attention: (i) controlling false positives, (ii) collinearity among explanatory variables, (iii) multiple comparisons and (iv) the choice of the tuning parameter that controls the amount of shrinkage and the sparsity of the estimates. The proposed techniques are relevant also in other contexts where LASSO is used for variable selection.

*Controlling false positives:* One approach for controlling false positives is to use Bayes factors. While marker-specific Bayes factors can be derived for models with indicator variables, including them in the shrinkage models can make the resulting Bayes factors suffer from a double shrinkage effect [5]. Also, the derivation of the posteriors of indicator variables afterwards in shrinkage models usually heavily depends on user-defined cut-off values to judge the QTLs [6]. Exceptions are shrinkage methods with coherent decision making frameworks such as [7] and [2] which both were able to derive Bayes factors without indicator variables as additional parameters in the model. In this paper, we instead control the false positives using the posterior distributions of the marker effects.

*Collinearity:* Collinearity among markers hinders the detection of an association by weakening the QTL signal. In association genetics, derivation of a significance threshold to judge the QTLs in the Bayesian LASSO has been suggested using (i) phenotype permutation [8, 9], (ii) Wald test statistic [10], or (iii) credible intervals [11]. All of them seem to suffer from collinearity between markers. Phenotype permutation works quite well but collinearity between markers weakens QTL signals by distributing parts of the signal over several markers. Wald test statistic does not work well in QTL detection since collinearity inflates the standard errors of the estimated effect sizes. Similarly, due to the bimodality of a QTL effect posterior caused by collinearity between adjacent markers, the credible interval of a true QTL often includes zero. To handle the problem of a weakened QTL signal caused by collinearity, combination of QTL signals over several adjacent markers has been suggested [12, 13]. We propose to take the potential collinearity among the markers into consideration by considering not only the credibility of each marker locus separately but also sums and differences of pairs of marker effects.

*Multiple testing:* There has been discussion about the need for multiple testing correction in Bayesian inference [14]. Bayesian perspectives on multiple comparisons are discussed also in [15]. One approach to correct for multiple testing is the Bayesian false discovery rate procedure (BFDR) [16]. However, BFDR requires that the posterior model includes a discrete indicator vector that defines the variable inclusions. If the posteriors for such indicator variables are derived afterwards, the definition of the cut-off values for variable inclusion is critical. We instead apply the method of highest point-wise probabilities (HPW) first described in [17] that detects variables with high enough joint posterior probability for effects and needs no extra user input in the inference.

*The choice of the tuning parameter:* Bayesian LASSO has a single shrinkage or tuning parameter that controls both the sparsity of the model (number of variables to be selected) and the amount of shrinkage applied to all effect coefficients. This tuning parameter may be considered either fixed [18] or random with its own prior [3]. The latter approach seems to be more common in practice. It corresponds to numerically integrating over the parameter and while analytical integration has also been suggested, it is reported to result in increased sensitivity to the hyperprior parameters [19]. If the tuning parameter is fixed, choosing an appropriate value with a criterion such as BIC or cross-validation has been suggested in [20]. Use of BIC leads to a sparser model than cross-validation which measures predictive performance. A bootstrap procedure of choosing the tuning parameter has also been suggested [21]. In a genetics context a different approach has been suggested where the relationship between heritability and the tuning parameter is derived under simplified conditions [22, 23] and then used to guide tuning parameter selection for traits with known heritability. In the case of prediction the performance of random tuning parameter was compared to the results obtained when a fixed tuning parameter was selected with cross-validation in [24]. Their results obtained using a random tuning parameter and using the best fixed value were close to each others.

**Fig 1:**
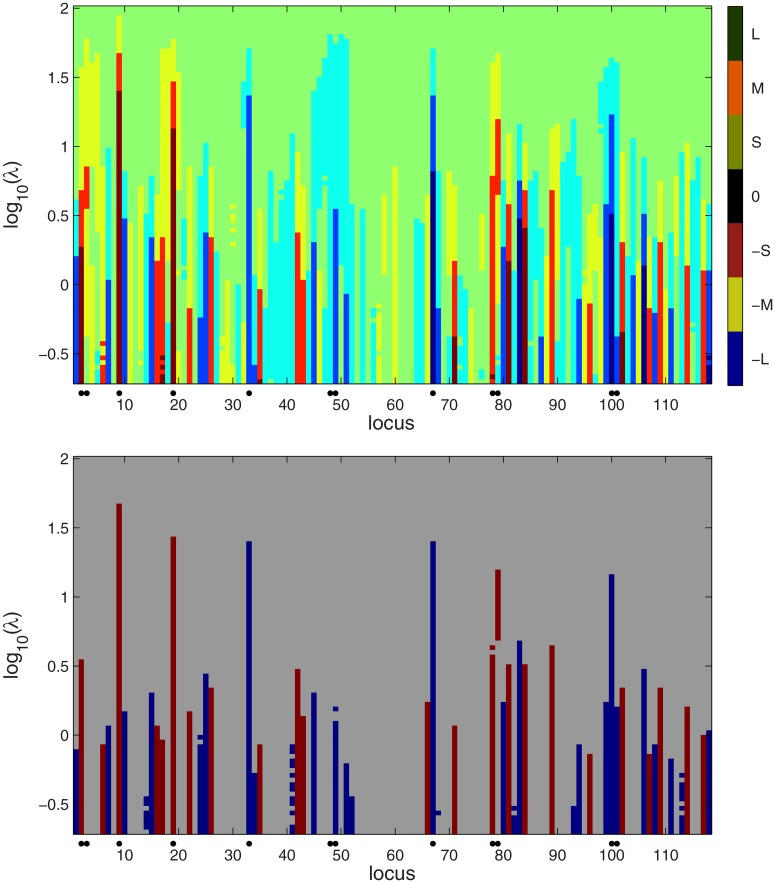
Scale space view of the Bayesian LASSO for the simulated Barley data. Upper panel: the quantized estimated posterior mean of *β* for a range of tuning parameters *λ*. Color categories: setting *B* = max_*j*_∣*β*_*j*_∣, 0: $|\beta_{j}|<\frac{B}{20}$, S: $\frac{B}{20}<\beta_{j}<\frac{B}{5}$, M: $\frac{B}{5}<\beta_{j}<\frac{2B}{5}$, L: $\frac{2B}{5}<\beta_{j}\leq B$. -S, -M, -L: similarly. Lower panel: credibility analysis to judge QTLs. For a given *λ*, the loci with credibly positive or negative QTL effects are flagged red or blue, respectively, and rest of the loci are gray. The locations of true QTLs are indicated with black dots in the bottom part of the maps. As some of the markers with a non-zero effect were removed, the flanking loci are indicated with black dots.

The estimation and decision making results depend heavily on the value of the tuning parameter. Therefore different conclusions might be made about the coefficients depending on the method to choose the tuning parameter. Here, instead of setting the tuning parameter random or estimating it from the data, we apply the idea of a scale space and consider a whole range of tuning parameter values in our analyses. The scale space concept has its origins in computer vision research (see for example [25]). The basic idea of scale space is that an object is viewed on several scales or resolutions. The smallest scales correspond to a microscopic vision where the finest details of the object are revealed, whereas the large scales correspond to a macroscopic vision where only the coarse features remain. Since its introduction to statistics, scale space has been extensively used in the analysis of data from various fields of research which however so far have not included any genetic applications [26, 27]. When scale space analysis is applied to Bayesian LASSO, a large value of the tuning parameter provides the macroscopic view in which one is able to identify only well-defined QTL regions that remain after a large number of marker effects are shrunk to zero. On the other hand, a small value of the tuning parameter provides the microscopic view that shows other, small scale features, corresponding to regions that can appear because a small value of the tuning parameter makes only a small number of marker effects shrink to zero. In the context of decision making, larger tuning parameter values can lead to false negatives in QTL detection, since a large number of SNP effects is ‘forced’ to zero. Similarly, small tuning parameter values can produce false positive QTLs, since a large number of markers now have their effects considered for detection. Inference methods such as HPW together with a scale space approach can provide a general and comprehensive way to control false positive and negative rates. Such an approach also helps to assess the sensitivity of the model to the choice of the tuning parameter and provides a means to suggest reasonable choices to use in the data analysis. As usual in statistical scale space methods, we visualize our results using color maps. In these scale space maps the horizontal axis represents the loci, the vertical axis represents the logarithm of the tuning parameter and the color at each pixel represents either the credibility or the posterior mean of the coefficient at a locus (cf. Fig. 1).

The importance of exploring a range of tuning parameter values is also emphasized in the solution path of LASSO [28] and in Bayesian sparsity-path-analysis [29]. In these approaches the results are visualized by drawing in same plot the estimated effect or the predictive relevance of each loci as a function of the tuning parameter. These plots and our scale space maps both provide information about the influence of the tuning parameter on the coefficients. The advantage, however, of using the scale space maps is that the impact of the tuning parameter on a given markers as well as the QTL candidates can be seen at a glance, whereas in the solution path type of plots, the markers can not be directly identified.

LASSO and ridge regression are similar penalized regression methods that differ only in the form of the penalty used. Ridge regression has been criticized for not being able to shrink the regression coefficients to zero and therefore being unable to perform proper variable selection. Likewise, ridge regression is considered to suffer from correlation between covariates, distributing effects between collinear variables. In addition to LASSO, we therefore included in our tests also Bayesian ridge regression.

The rest of the paper is organized as follows. In Section 2.1, we give a short overview of LASSO and the Bayesian LASSO. Section 2.2 explains the basic idea of our approach and introduces some of the graphical tools used in the analyses. Section 2.3 discusses methods for handling collinearity and multiple testing. These methods can be used with a fixed or a random tuning parameter. The scale space view is taken up again in Section 2.4 where the decision making in scale space is discussed. The ideas suggested in this paper are applied to Bayesian ridge regression and classic LASSO in the on-line supplement S1 Appendix. The feasibility of the methods is demonstrated with three data sets in the Results section. First, in Subection 3.1, we describe the suggested procedures in more detail using a simulated genetic data set. A larger simulated data set is analyzed in Subsection 3.2 and a real dataset is analyzed in Subsection 3.3. Finally, the last section contains a summary and a discussion of the results.

## Methods

### 2.1 LASSO and Bayesian LASSO

Consider the regression model

$$\begin{array}{c} y=\mu 1+X\beta +\varepsilon , \end{array}$$ (1)

where **y** = [*y*_1_,…,*y*_*n*_]^*T*^ is the *n*×1 response vector, *μ* is the overall mean, **X** = [*x*_*ij*_] is the *n*×*m* design matrix of standardized regressors, *β* = [*β*_1_,…,*β*_*m*_]^*T*^ is the *m*×1 vector of regression coefficients and **ε** = [*ɛ*_1_,…,*ɛ*_*n*_]^*T*^ is an *n*×1 zero mean Gaussian i.i.d. noise vector with variance *σ* ^2^.

In LASSO regression of [30], the estimates of the regression coefficients are obtained using *L*_1_-constrained least squares. Thus,

$$\begin{array}{c} \hat{\beta }=\underset{\beta }{\text{arg}\;\text{min}}\left({∥y-\mu 1-X\beta ∥}^{2}+\lambda \sum_{j=1}^{m}\left|\beta_{j}\right|\right). \end{array}$$ (2)

Here *λ* ≥ 0 is a tuning parameter that controls the weight of the penalty term in the penalized least squares. The larger the value of *λ*, the sparser the estimate of *β* is [30].

The minimizer of (2) is actually equal to the posterior mode of the model (1) when the regression coefficients in *β* are assumed to have independent Laplace priors [30]. Laplacian distribution can be fitted either nonhierarchically [31] or hierarchically using so-called scale-mixture parametrization [32]. In the Bayesian LASSO, the scale-mixture parametrization is more often used as it provides straightforward and transparent conjugacy properties and easier implementation [33]. Park and Casella [3] used a conditional Laplace prior of the form

$$\begin{array}{c} p\left(\beta |\sigma^{2}\right)\propto \prod_{j=1}^{m}e^{-\lambda |\beta_{j}|/\sqrt{\sigma^{2}}} \end{array}$$ (3)

with the scale-mixture approach and obtained the hierarchical model

$$\begin{array}{ccc} y|\mu ,X,\beta ,\sigma^{2} & ∼ & N(\mu 1+X\beta ,\sigma^{2}I),\\ \beta |\sigma^{2},\tau_{1}^{2},\cdots ,\tau_{m}^{2} & ∼ & N(0,\sigma^{2}D_{\tau }),\\ D_{\tau } & = & \text{diag}(\tau_{1}^{2},\cdots ,\tau_{m}^{2}),\\ p(\sigma^{2},\tau_{1}^{2}\cdots ,\tau_{m}^{2}) & \propto & p\left(\sigma^{2}\right)\prod_{j=1}^{m}\frac{\lambda^{2}}{2}e^{-\lambda^{2}\tau_{j}^{2}/2},\\ \sigma^{2},\tau_{1}^{2},\cdots ,\tau_{m}^{2} & > & 0. \end{array}$$ (4)

For *σ* ^2^, they suggested an uninformative prior, *p*(*σ* ^2^) ∝ 1/*σ* ^2^, or any inverse-gamma prior and for *μ*, a flat prior was proposed. In the prior (3), *λ* is scaled with *σ* ^2^ to guarantee a unimodal full posterior distribution. Park and Casella proposed to either estimate the tuning parameter *λ* using marginal maximum likelihood or to set a gamma prior for *λ* ^2^.

### 2.2 A scale space approach

In nonparametric regression, the signal underlying noisy data **y** is often estimated by a smooth **S**_*λ*_ **y**, where **S**_*λ*_ is a smoothing operator and the smoothing parameter *λ* > 0 controls the level of smoothing applied. Consider for example time series data

$$\begin{array}{c} y=\mu +\varepsilon , \end{array}$$

where **μ** is the underlying truth and **ε** is noise. An example of a smoothing operator is the discrete spline smoother defined by

$$\begin{array}{c} S_{\lambda }y=\underset{u}{\text{argmin}\;}\left\{{∥y-u∥}^{2}+\lambda u^{T}Qu\right\}={\left(I+\lambda Q\right)}^{-1}y, \end{array}$$ (5)

where **Q** = **C** ^*T*^ **C** and **Cu** is the vector of second differences of **u** (see [17, 34, 35]). The traditional procedure is to look for a *λ* for which **S**_*λ*_ **y** ≈ **μ**. This can be difficult and the scale space approach sidesteps the problem by considering all smoothing levels, with each smooth thought to provide valuable information about **μ** at a particular scale. The effect of the smoothing parameter on the data in a similar context is visualized e.g. in the top panel of Fig. 2 in [36].

**Fig 2:**
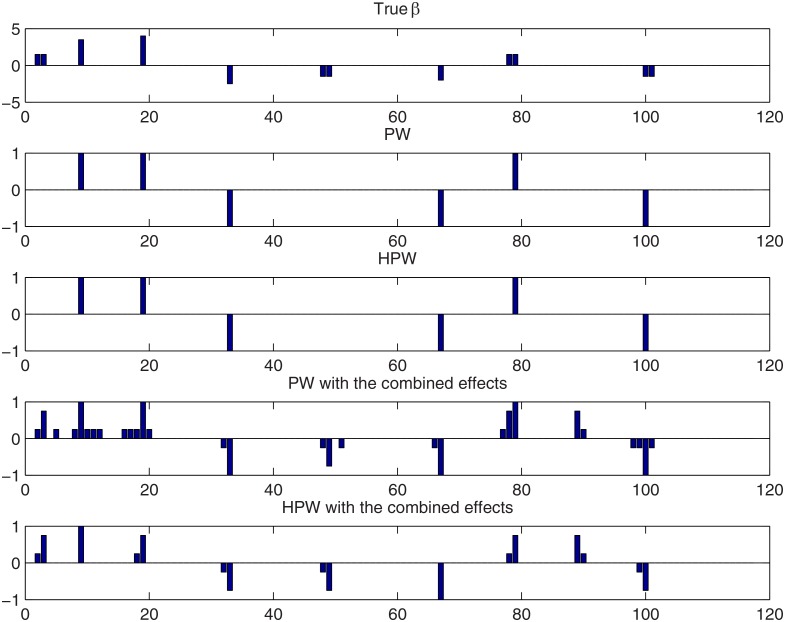
QTL credibility analysis of simulated Barley data with the Bayesian LASSO using a random **λ**. Top panel: the true effect vector *β*. Second and third panels: the loci with credibly non-zero effects detected using point-wise and simultaneous inference, respectively. Here 1 corresponds to a credibly positive locus and -1 corresponds to a credibly negative locus. Fourth and fifth panels: the loci with credibly non-zero coefficients detected using point-wise and simultaneous inference when also combined effects are considered. See the text for the interpretation of the bar heights.

Clearly, the role of the tuning parameter *λ* is similar in (2), (4) and (5) in that a small penalty (small *λ*) produces an estimate with many details (variables), whereas a large penalty (large *λ*) results in a smooth or sparse estimate with fewer details. We therefore propose a scale space version of LASSO and the Bayesian LASSO where, instead of a single tuning parameter, a whole range of *λ*’s is considered. And, just as in other statistical scale space methods (e.g. bottom panel of Fig. 2 in [36]), the results are visualized in color maps that summarize the results of the analyses. An example of such a map is the upper panel Fig. 1 with each row showing the posterior mean of *β* for the value of *λ* indicated on the vertical axis.

In addition to estimating the coefficient vector *β*, we also want to detect the individual regression coefficients *β*_*j*_ that have a credible effect on the response variable. This is done by analyzing the posterior distribution of *β* for each *λ*. In association genetics one typically considers the marginal posterior distributions of the individual regression coefficients *β*_*j*_ separately and detects loci *j* where the (1−*κ*)-level credible interval of *β*_*j*_ does not contain zero (cf. [11]), and where often the value *κ* = 0.05 is used. This is achieved by finding the loci where ℙ(*β*_*j*_ > 0∣**y**) > *α* or ℙ(*β*_*j*_ < 0∣**y**) > *α*, with *α* = (1−*κ*/2) = 0.975. In practice, this is done by indentifying the loci where the proportion of positives or negatives in the posterior sample exceeds 0.975. We call such marginal inference point-wise (PW) as it only considers one locus at a time. The results are again visualized using a map, an example of which is shown in the lower panel of Fig. 1. We believe that scale space analyses such as those summarized in Fig. 1 help to determine a reasonable value for *λ* that then can be used in making decision about interesting associations in the data. They also inform the analyst about the sensitivity of the results to the choice of the tuning parameter.

For LASSO, Tibshirani [30] visualized the dependence of *β* on the tuning parameter by so-called trace plots, drawing in the same plot the effects *β*_*j*_ as a function of *λ* for all *j* = 1,…,*m*. In solution path of LASSO [28] and Bayesian sparsity-path analysis [29] similar plots were also used to visualize the dependence of the effects on the tuning parameter. While both trace and solution path plots and our scale space maps all shed light on the effect of the tuning parameter on the estimated regression coefficients, the advantage of scale space maps is that they enable the identification of the possible QTL loci at a glance whereas in solution path type of plots the QTL’s cannot be directly identified.

To increase the usefulness of the proposed methods, three problems need to be addressed. First, a small value of *λ* produces a large number of false positives, that is, coefficients *β*_*j*_ that erroneously appear as credibly non-zero. This is due to the fact that when *σ* ^2^ is treated as a random variable, its posterior distribution depends on the value of *λ*. When the number of explanatory variables is about the same as the number of observations and *λ* is small, the posterior mean 𝔼(*μ* **1**+**X** *β*∣**y**) resembles the data **y** and the posterior mean of *σ* ^2^ is small, which produces a lot of false positives. In a scale space map, QTL effects can be identified by a color bar that persists through a wide range of *λ*’s. This approach is based on an assumption that the effect sizes of the most important markers are substantially larger than those of the unimportant ones and that therefore increasing *λ* shrinks them to zero more slowly than markers with no effect. A threshold for *λ* can be set by the eye by finding a value for which only the strongest effects are credible. In Section 2.4 we propose a more formal, phenotype permutation based, approach for limiting *λ*. As a different approach we also propose to let the credibility level *α* depend on *λ* and then determine *α*(*λ*) using phenotype permutations.

Second, if the explanatory variables are collinear, PW inference has difficulty in detecting the important variables among all explanatory variables because an effect may be distributed over several collinear variables so that none of them is detected separately. In Section 2.3.1 we propose to solve this problem by considering not only individual effects but also their functionals (sums and differences). Finally, there is the problem of “multiple comparisons” when only the marginal posterior distributions are considered in the inference. This problem is addressed in Section 2.3.2.

### 2.3 Collinear explanatory variables and inference under multiple testing

The methods that we propose for handling collinearity and inference for multiple testing are not tied to the scale space view and may therefore be used also in the traditional setting where the tuning parameter *λ* is either set random or estimated from the data.

#### 2.3.1 Posterior inference for correlated regressors

The most straightforward approach to collinearity of variables **x**_*j*_ and **x**_*k*_ is to consider the proportion of posterior samples where at least one of the coefficients *β*_*j*_ or *β*_*k*_ is positive/negative. This approach is particularly applicable with methods in which inclusion of a locus *j* in the model is indicated by an auxiliary indicator variable (slab-and-spike) but in principle it can be used in other contexts, too. Collinearity is handled with such an approach e.g. in [12] using stochastic search variable selection (SSVS) [37].

The problem with applying such an approach in the Bayesian LASSO or ridge regression is that the posterior probability of an effect depends on the correlation between the explanatory variables. This is of course undesirable and we propose to estimate the combined effect of **x**_*j*_ and **x**_*k*_ using the posterior distribution of *β*_*j*_+*β*_*k*_ or *β*_*j*_−*β*_*k*_. This can be justified as follows. The combined effect of **x**_*j*_ and **x**_*k*_ is *β*_*j*_ **x**_*j*_+*β*_*k*_ **x**_*k*_ and with the genetic data sets considered here, the possible values of *x*_*lj*_ and *x*_*lk*_ are 1 and -1. Therefore, if *x*_*lj*_ = *x*_*lk*_ the combined effect of **x**_*j*_ and **x**_*k*_ on the observation *y*_*l*_ depends on the sum *β*_*j*_+*β*_*k*_. It may, however, happen that the direction of indicator variables of a group of collinear loci may be opposite in which it is supposed to fit [38, 39]. Therefore, we also consider the difference *β*_*j*_−*β*_*k*_. To reduce the number of combined effects one needs to analyze, we choose a threshold 0.5 < *ρ* < 1 and for *β*_*j*_+*β*_*k*_ only test pairs for which the proportion of observations with *x*_*lj*_ = *x*_*lk*_ exceeds *ρ* and for *β*_*j*_−*β*_*k*_ we only test pairs for which the proportion observations with *x*_*lj*_ ≠ *x*_*lk*_ exceeds this limit. In all our examples we took *ρ* = 0.8. In the following we will denote by *δ*_*jk*_ a combined effect, either *β*_*j*_+*β*_*k*_ or *β*_*j*_−*β*_*k*_. The posterior distribution of *δ*_*jk*_ is used to compute a credible interval and the combined effect is considered credible if the interval does not contain zero.

Note that we do not require that the combined effects consist of neighboring loci. This is an important point as the markers that are members of the same biological pathway could be highly correlated but their chromosomal positions do not have to be physically close to each other. Also, if needed, the method could easily be extended to handle combinations of three or more effects. If one tests only the combinations where the pairwise proportion of *x*_*lj*_ = *x*_*lk*_ (or *x*_*lj*_ ≠ *x*_*lk*_) is higher than *ρ*, the number of combinations should not explode, given that the value of *ρ* is high enough.

#### 2.3.2 Simultaneous inference

We adopt here the method of highest point-wise probabilities (HPW) suggested in [17]. Collecting the combined effects into a vector **δ** and denoting **γ** = [*β* ^*T*^,**δ** ^*T*^]^*T*^, HPW selects the components of **γ** in an order of descending marginal (PW) probability as long as the joint probability of positivity or negativity of the selected effects exceeds the desired credibility level *α*. We use the value *α* = 0.95. Here we modify this procedure so that *γ*_*s*_ = *β*_*j*_ will not be included if a combined effect containing *β*_*j*_ has already been selected. Also, a combined effect *γ*_*s*_ = *δ*_*jk*_ cannot be selected if *β*_*j*_, *β*_*k*_, or *δ*_*pq*_ has already been selected and *j* or *k* belongs to the set {*p*,*q*}.

The strength of the HPW method is that the detection of the most important loci does not depend on the number of tested insignificant coefficients or combined effects, which is the case for example with permutation tests. Hence, the few most important loci are equally detectable among hundreds or thousands of dispensable coefficients.

### 2.4 Decision making and scale space

In this section we propose two alternative ways to handle the multiple false positives with small values of the tuning parameter.

#### 2.4.1 Limiting the tuning parameter value

**Fig 3:**
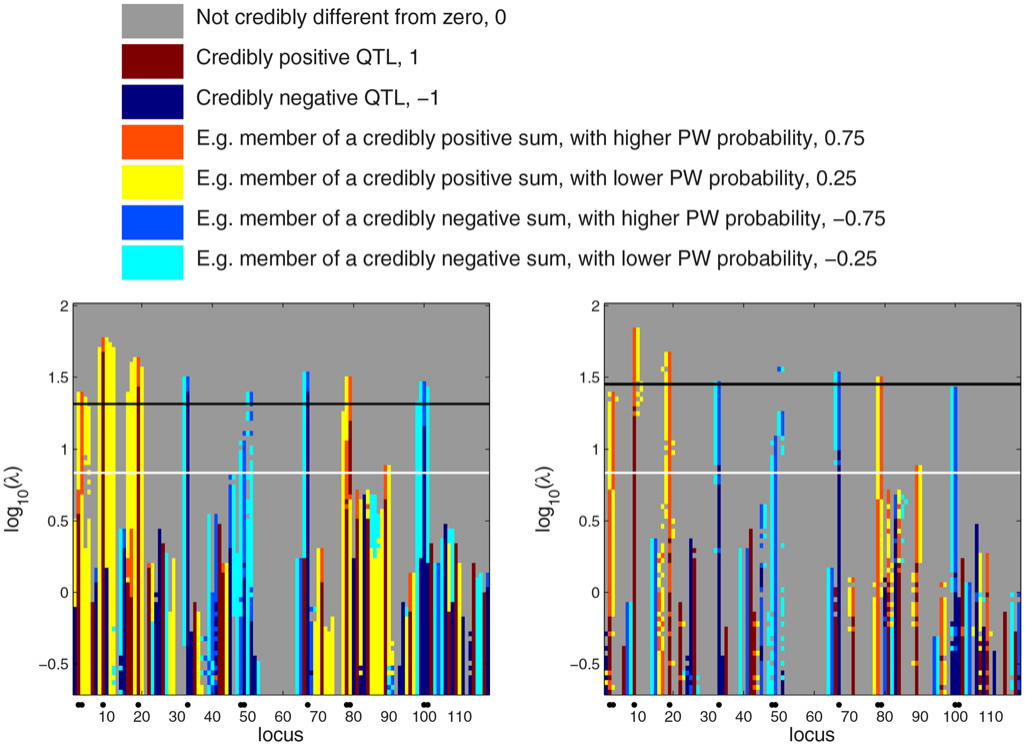
Scale space QTL credibility analysis of the simulated Barley data. Left: PW map with combined effects. Right: HPW map with combined effects. The white horizontal line marks the posterior mean of the tuning parameter *λ*. The black line marks the phenotype permutation based threshold for *λ*. The precise significance of the colors follows the conventions adopted in Section 3.1.2, where each associated numerical value corresponds to a particular credible instance of a combined effect. These numerical values also determine the bar heights in illustrations such as Fig. 2.

In Section 2.2 we argued that the number of false positives can be reduced by selecting a *λ* threshold where only the strongest effects are credible. In addition to such a visual inspection, a more formal rule for limiting *λ* procedure can be adopted. For this, we deviate from the Bayesian paradigm and propose to use a phenotype permutation based approach to limit the values *λ* considered in detection (see [8]). A limit is obtained separately for PW and for HPW inferences as follows. Credibility maps such as the one in Fig. 3 are constructed repeatedly using hypothetical data sets where the phenotype vector **y** is randomly permuted under the null hypothesis of no phenotype-marker association. The limit of *λ* is then set to the level where 95% of the repetitions produce no false detections.

#### 2.4.2 Connecting credibility level and the tuning parameter

One can also control the number of false positives that appear for small values of *λ* by adjusting PW inference so that, depending on *λ*, the credibility threshold *α* can have a higher value than the usual 0.975. Again, phenotype permutation will be used (cf. [12]). For each *λ* we permute repeatedly the phenotype vector **y** randomly. Then, for a fixed *λ* and a fixed permutation, we compute the maximum of the marginal posterior probabilities of the effects and the combined effects of being either positive or negative. The value *α*(*λ*) is then the 0.95 quantile of this maximum when all permutations are considered. For a given *λ*, the effects and combined effects for which the marginal probability exceeds max{*α*(*λ*),0.975} are then flagged as credible. Despite the fact that only marginal probabilities are used, this procedure can be viewed as a version of simultaneous inference because maximum taken over the individual probabilities is used to set the credibility level.

## Results

### 3.1 An illustrative example

We demonstrate the scale space approach in the Bayesian LASSO using an artificial genetic data set with *n* = 145 observations and *m* = 127 explanatory variables. The design matrix **X** = [**x**_1_,…,**x**_*m*_] is taken from the well-known North American Barley data set published in [40]. This is an experimental dataset arising from a line crossing experiment. Each entry of **X** has two possible values, 1 or -1. We did not standardize the **x**_*j*_ vectors. However, some values in the original matrix **X** were missing and we treated them as follows. Five loci (variables) that contained a considerable number of missing values (from 33% to 55%) were removed from **X**. In all the remaining loci the percentage of missing values was less than 20 and on average 3.4. The missing values in the remaining loci were imputed once at random with equal probability for 1 and -1. The associated QTL effect vector *β* is shown in Table 1. Gaussian i.i.d. noise with standard deviation 8 was added to **X** *β* to obtain **y** which then represents the observed phenotypes. The heritability in the data, that is, proportion of phenotypic variance attributable to genetic factors, Var(**X** *β*)/Var(**y**), was approximately 0.89.

**Table 1:** The non-zero components of the QTL effect vector *β* for the simulated Barley data.

Original locus	Adjusted locus	Simulated effects *β*_*j*_
3	(2,3)	3
10	9	3.5
20	19	4
34	33	-2.5
51	(48,49)	-3
72	67	-2
84	(78,79)	3
108	(100,101)	-3

The original and the adjusted loci indicate the locations of the QTL effects before and after modifying the data (i.e. after removing the five markers with considerably many missing values and the four QTL markers). As some of the markers with a non-zero effect were removed, the flanking loci are indicated in the middle column.

One of the difficulties in genetic marker detection is that usually the QTL is located between two markers. As the neighboring marker patterns are usually correlated, such an effect needs to be found using the flanking loci. In this data set also, the correlation between the genotype patterns **x**_*j*_ and **x**_*j*+1_ is high in adjacent loci *j* and *j*+1. In order to emphasize the impact of collinearity when the true genotypes of the QTL cannot be observed, four of the eight QTLs were removed from **X** before the analysis (see Table 1). The high heritability in the complete data might suggest an easy detection task. However, as four QTLs have been removed from the data prior the analysis, only partial QTL information is left in the data and carried by the closest flanking markers.

In Section 3.1.1 we show the basic scale space analysis highlighting the problem of numerous false positives obtained with small tuning parameter values and the need to take the collinearity into account. In Section 3.1.2 we demonstrate our solutions for collinearity and multiple testing, for the sake of simplicity, first using a random tuning parameter. Finally, in Section 3.1.3 we return to the scale space setting and demonstrate the use of our tools for collinearity, multiple testing and exclusion of the false positives produced with a small value of the tuning parameter.

#### 3.1.1 The basic scale space analysis

As suggested in [3], an uninformative prior *p*(*σ* ^2^) ∝ 1/*σ* ^2^ was used for the noise variance. The posterior sample was drawn from (4) using the R-package BLR [41]. To be able to obtain the marginal and joint posterior probabilities of the coefficients, we modified the BLR-function so that it returns the posterior samples. The modified code can be downloaded from http://cc.oulu.fi/~lpasanen/SSBLASSO and lib.stat.cmu.edu/general/SSBLASSO.zip. After a burn-in period of 2.5⋅10^4^ iterations, we kept every 4th sample until 10^4^ samples were obtained. For scale space analysis, the *λ*-range included 80 values from 10^−0.7^ to 10^2^ with logarithmic spacing.

The posterior mean of *β*_*j*_ at each locus *j* and for each *λ* is displayed in the upper panel of Fig. 1. For clarity, the effect sizes were quantized to 7 levels. The QTLs are indicated with black dots in the lower part of the map. Large absolute values of the *β*_*j*_’s are indicated by the pattern of strong colors in the lower part of the map. This is expected because the penalty for large ∣*β*_*j*_∣ is mild for a small *λ*. Around *λ* = 10^1^ most of the actual QTL effects clearly differ from zero while the rest of the *β*_*j*_’s appear to have shrunk close to zero. When *λ* > 10^2^ all coefficients have become small.

The credibility map is shown in the lower panel of Fig. 1. In this map, each row summarizes the credibility of the vector *β* for a given value of *λ*. Here credibility is assessed point-wise, treating each locus separately and identifying the coefficients whose 95% posterior credible interval does not contain zero. Red and blue flag the credibly positive and negative coefficients. There is a large number of false positives for *λ* < 10^−0.25^. A threshold for *λ* can be set by the eye by finding a value for which only the strongest effects are credible. In the credibility map of Fig. 1, most false positives disappear while the actual QTLs remain credible when *λ* > 10^0.5^. A suitable threshold for *λ* might therefore be for example *λ* = 10^1^.

In addition to false positives, another concern is the difficulty to detect effects in the presence of collinearity among loci genotype patterns **x**_*j*_. In this example, when *λ* > 10^0.5^, the effect at the locus (48,49) is not credible and the effect at (2,3) is barely so when *λ* > 10^0.5^ and therefore both would be missed if a limit *λ* = 10^1^ would be applied. The map of posterior means (upper panel of Fig. 1) shows that the effect at (48,49) has been distributed over several neighboring loci none of which is strong enough to be detected in the credibility map unless *λ* is well below 10^0.5^.

#### 3.1.2 Analysis with a random tuning parameter

In this section we illustrate the performance of our methods for handling collinearity and simultaneous inference using random tuning parameter. We use *λ* with the improper uninformative prior *p*(*λ*) ∝ 1/*λ* and a random *σ* ^2^ with the improper prior *p*(*σ* ^2^) ∝ 1/*σ* ^2^. A sample of size 1.5⋅10^4^ from the posterior distribution of *β* was drawn by keeping every 10th sample after a burn-in period of 7⋅10^4^ iterations. Fig. 2 displays the credibly positive and negative effects obtained with different approaches. The top panel in Fig. 2 presents the underlying true *β*. As the original trait loci (3, 51, 84, 108) were removed from the data, their associated effects are in this plot divided equally between the two flanking loci. The rest of the panels show the loci detected by PW and HPW inferences with and without combined effects. The bar heights do not reflect the actual effect sizes but only the type of credibility inferred at each locus as follows. In PW and HPW analyses without combined effects, the values 1 and -1 indicate a locus with a credibly positive and credibly negative effect, respectively. When also combined effects are considered, 1 at a locus *j* indicates that *β*_*j*_ is credibly positive whereas 0.75 and 0.25 indicate that at least one of the following is true: a sum *δ*_*jk*_ (or *δ*_*kj*_) is credibly positive or a difference *δ*_*jk*_ is credibly positive, (or the difference *δ*_*kj*_ is credibly negative). Here the value 0.75 indicates that the effect *β*_*j*_ is the member of the two effects involved in *δ*_*jk*_ (or *δ*_*kj*_) with the higher marginal posterior probability of being positive or negative and the value 0.25 indicates that *β*_*j*_ is the effect with the lower marginal probability. If more than one of these conditions hold at a locus *j*, the condition that results in the highest bar height is applied. The interpretation is analogous for the negative values -1, -0.75 and -0.25.

As indicated by the upper map of Fig. 1, collinearity spreads the effects at the loci (48,49) and (2,3) over their neighborhoods and Fig. 2 shows that these loci are in fact not detected unless combined effects are included in the analysis. We also notice that a false positive sum *β*_89_+*β*_90_ is detected both by PW and HPW inference. This is due to the fact that the posterior of the tuning parameter *λ* favors slightly too small values with this data set.

For these data, point-wise and simultaneous inference produce rather similar results. If combined effects are not considered, the results are in fact identical. When the combined effects are included, several loci near the QTLs are detected by PW inference because for QTL locus *j*, any combined effect involving *β*_*j*_ can be credibly positive or negative. The best candidate for a QTL then is the locus with the larger marginal probability, that is, the locus where the height of the bar is either 0.75 or 1. With simultaneous inference (HPW), only the strongest sums or effects are detected.

#### 3.1.3 Improved Scale space analysis

Fig. 3 presents the scale space view of PW and HPW inferences when also combined effects are considered. For small *λ*, many false positives appear, even with simultaneous (HPW) inference. In Section 3.1.1 we suggested that the QTLs could be detected as the highest peaks in PW and HPW maps. In both maps of Fig. 3 the QTLs indeed can be detected without false alarms provided that inference is limited to rows where *λ* > 10^1^.

**Fig 4:**
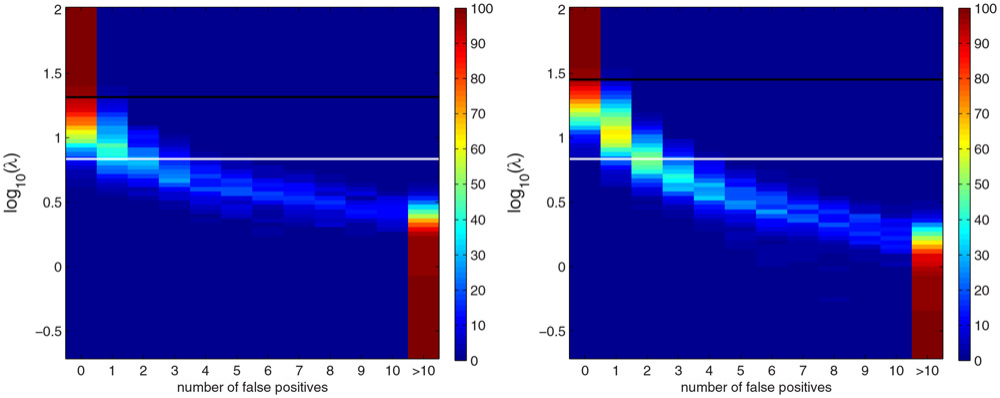
The distribution of the number of false positives for simulated Barley data under the null hypothesis of no effects. Each row represents the distribution of the number of false positives in 100 samples generated under the null hypothesis with a fixed value of *λ*. Left and right panels represent the results obtained with PW and HPW inference, respectively. The white horizontal line marks the posterior mean of the tuning parameter *λ*. The black line marks the *λ*-level where 95% of the repetitions produce no false detections.

Next we will consider a phenotype permutation based threshold for *λ*. The summaries of 100 repetitions using PW and HPW inference are shown in Fig. 4. In the maps, each row represents the distribution of the number of false positives at a given value of *λ*. As one QTL may create several credibly non-zero combined effects, the number of false positives here is defined as the number of loci with “credibility type” 1, -1, 0.75 or -0.75 (cf. Section 3.1.2). For small values of *λ* all repetitions contain at least 10 false positives. The distribution changes rapidly when *λ* is in the interval [10^0^,10^1^] so that at least 95% of the repetitions have no false positives at *λ*_PW_ ≈ 10^1.32^ for PW inference and at *λ*_HPW_ ≈ 10^1.45^ for HPW inference. These levels are marked with black lines in Fig. 3. In PW inference, the peaks at all QTL positions exceed *λ*_PW_, whereas for HPW the limit *λ*_HPW_ is rather conservative as three effects do not quite reach it. However, the missing locus (48,49) is discovered anew when *λ* > 10^1.5^ and therefore can be considered as being detected. For comparison, the posterior mean of *λ* is marked with a white line. It is well below the thresholds obtained by permutations. This is in line with the fact that in Section 3.1.1, PW and HPW inferences with a random *λ* detected all the QTLs but produced also one false positive (cf. Fig. 2).

We observed in Section 3.1.1 that PW inference detected all combined effects that involve a true QTL. For the same reason, the peaks in a PW map such as the left panel of Fig. 3 are wide. For example, the QTL at locus 9 is detected in sums *β*_9_+*β*_*k*_, *k* = 8,10,11,12. However, in all these sums, the marginal posterior probability is higher at the locus 9, the rest of the loci being weaker members of the sum. The best QTL candidate is therefore locus 9. The peaks in the HPW map (right panel of Fig. 3) are thin since only the strongest sum or an individual effect is detected. For example, only the sum *β*_9_+*β*_10_ is flagged as credible, with locus 9 being the stronger member.

**Fig 5:**
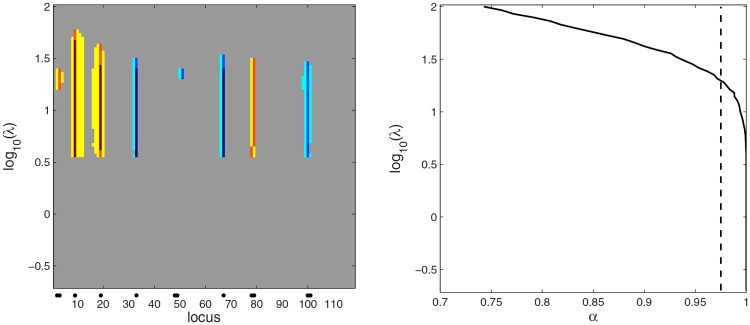
Using a λ-dependent credibility threshold for the simulated Barley data. Left: QTL credibility analysis using PW inference with a *λ*-dependent credibility threshold. For color coding see the caption of Fig. 3. Right: The dependency between *α* and *λ* with the limit *α* = 0.975 marked with a dashed line. See the text for more information.

Second, we consider the approach where the credibility level is let to depend on the tuning parameter. The scale space map using this procedure with 100 random permutations is displayed in the left panel of Fig. 5. The color coding is the same as in an ordinary PW map with combined effects (cf. Fig. 3). The function *α*(*λ*) is displayed in the right panel with the limit *α* = 0.975 shown with a vertical dashed line. For small *λ*, *α*(*λ*) = 1 and none of the QTLs are detected. As *λ* increases, *α*(*λ*) decreases and consequently the marginal posterior probability of a positive or negative effect decreases at loci with zero true effect. At the same time, the posterior probability of a non-zero effect for true QTLs hopefully remains high. In the present example, every QTL is detected with at least one value of *λ*.

#### 3.1.4 Comparisons

**Fig 6:**
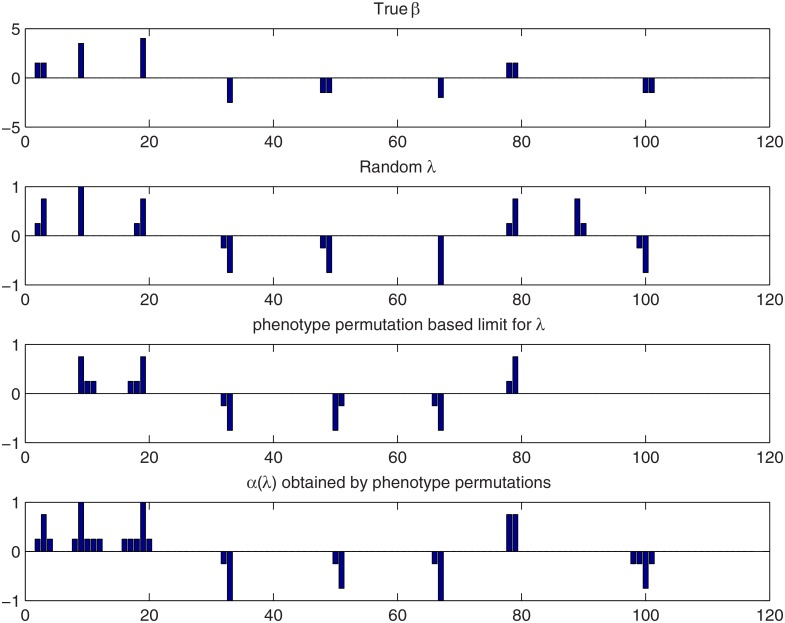
Comparison of detection methods for the simulated Barley data when the Bayesian LASSO is used. Top panel: the true QTL effect vector *β*. Second panel: the results for HPW inference with a random *λ*. Third panel: the results for HPW inference by limiting *λ* using phenotype permutation. Bottom panel: results for the *λ*-dependent credibility threshold. See Section 3.1.2 for the interpretation of the bar heights.

Detection results for the two methods proposed in Section 2.4 as well the standard approach of treating *λ* as random are compared in Fig. 6. True effects are displayed in the top panel. The second and third panel show the results of HPW inference using random *λ* and *λ* ≥ *λ*_HPW_, respectively. In the third panel, the bar height at a locus is set to the maximum absolute “credibility type” value over all values of *λ* ≥ *λ*_HPW_ in the HPW scale space map (cf. Section 3.1.2 or caption of Fig. 3 where the numerical values associated with the credibility types are defined). The fourth panel shows the results for a *λ*-dependent credibility threshold *α*. The bar length at a locus *j* is now defined as the maximum absolute credibility type value over all *λ*’s in the *α*(*λ*) scale space map shown in left panel of Fig. 5. All the QTLs are detected, either by considering *λ* random or with PW inference, both by employing the lower bound *λ* ≥ *λ*_PW_ or a *λ*-dependent credibility level. However, random *λ* also produces one false positive sum effect. Using the lower bound *λ* ≥ *λ*_HPW_ seems to be more conservative and two QTLs are missed.

### 3.2 Simulated Wheat data

In our second example, the regression model design matrix **X** is the design matrix of Wheat data from International Maize and Wheat Improvement Center (CIMMYT) [42]. The data was obtained from the BLR R-package [41], where the markers with a minor allele frequency lower than 0.05 were first removed. In our experiments we used the 400×300 upper left corner of this matrix. Hence, there are *m* = 400 explanatory variables and *n* = 300 observations. These are more typical association data available for breeding populations. We used the coding -1 and 1 for the two genotypes without standardization. In contrast to the simulated Barley data, now neighboring loci are practically uncorrelated. There are several loci where the frequency of the value 1 is over 0.9 or less than 0.1. For meaningful inference, these loci were ruled out when QTLs were defined. The QTLs set for this simulated example are presented in Table 2. The observation vector **y** was obtained by adding Gaussian noise with standard deviation 8 to the **X** *β* to. Heritability for these data is approximately 0.77.

**Table 2:** The non-zero components of the QTL effect vector *β* for the simulated Wheat data.

Locus	Simulated effects *β*_*j*_	Locus	simulated effects *β*_*j*_
7	1.75	218	-2.25
76	2	273	-1.75
118	2.5	344	2.5
162	-2.25	395	-2.25
181	1.5		

**Fig 7:**
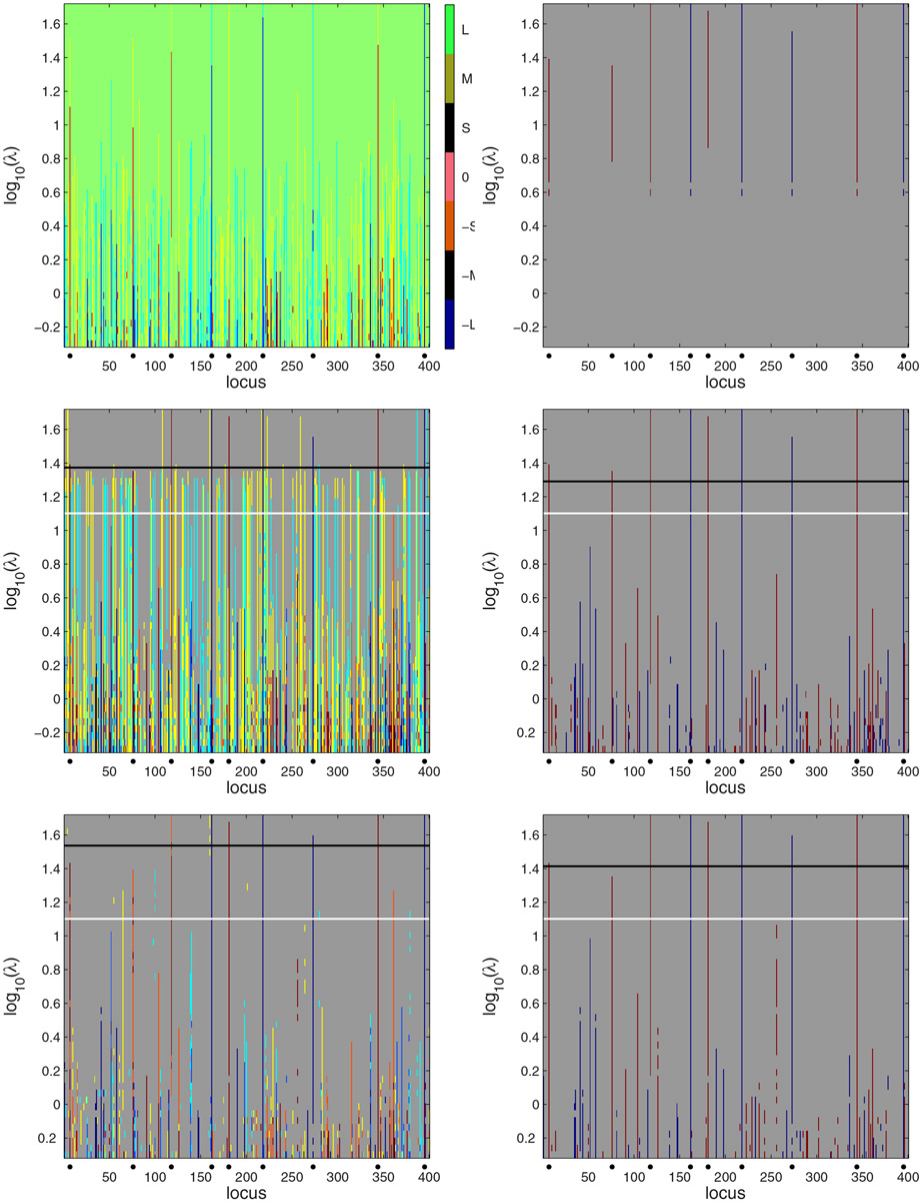
Scale space Bayesian LASSO QTL analysis of the simulated Wheat data. On left, from top to bottom: the quantized estimated posterior mean of *β* for a range of tuning parameters *λ*, QTL credibility analysis with PW and HPW inference. The white horizontal line marks the posterior mean of a random *λ*. The black line marks the phenotype permutation based threshold for *λ*. Right from top to bottom: the QTL credibility analyses without combined effects with a *λ*-dependent credibility threshold, PW inference and HPW inference. For color interpretation see captions of Fig. 1 and Fig. 3.

For posterior sampling we used the same setup as with the simulated Barley data set of Section 3.1. For scale space analysis we used 50 values of *λ* ranging from 10^−0.3^ to 10^1.7^ with logarithmic spacing. The scale space Bayesian LASSO map is shown in the top left panel of Fig. 7 and the corresponding PW and HPW credibility maps are in the middle and the bottom left panels. The phenotype permutation based limit for *λ* (obtained using 100 permutations) is marked with a black line and the posterior mean of a random *λ* is marked with a white line. Most of the QTLs stand out from false positives as higher peaks in the HPW map.

The high yellow and light blue peaks in the PW map are caused by the QTL at locus 76 where most (87%) observations have the value -1, and which therefore correlates with some of the other loci for which the frequency of 1 or -1 is high, making combined effects involving the locus 76 and such loci credible. However, in all of these false detections the correlated locus is the weaker member of a combined effect and therefore should not be considered as a QTL. This underlines the importance of considering only the member of combined effect with the higher marginal posterior probability as a QTL candidate. The numerous yellow and light blue false alarms could be eliminated by using a higher threshold than the value *ρ* = 0.8 employed now (cf. Section 2.3.1); for example, setting *ρ* = 0.9 would eliminate most of them. None of this is a problem for HPW inference that picks only the strongest effect involving a locus. All QTLs except for locus 76 exceed the phenotype permutation limit in PW inference. Also, only very few weaker effect members are falsely flagged as credible. Being more conservative, HPW inference misses the QTLs at the loci 7 and 76. At loci 7 and 76, the frequency of the values 1 and -1 is high (0.86 and 0.87, respectively). This great imbalance probably explains the difficulty of detecting these QTLs.

The PW and HPW maps obtained without considering the combined effects are shown in the middle and bottom right panels of Fig. 7. Now in the PW map all QTLs exceed the permutation based limit and for the HPW map this is the case for all QTLs except locus 76. The fact that the phenotype permutation approach now produces more liberal results stems from two facts. First, unlike for the simulated Barley data considered earlier, the effects now are not distributed over several loci and the QTLs can in fact be found directly from the posterior mean effects vector (top left panel of Fig. 7). Second, since combined effects were not included in the inference, the number of effects involved in phenotype permutations used was smaller which pushed the *λ*-limit lower. Locus 7 which was missed by combined effects analysis is now detected both by PW and HPW inference. The result with *λ*-dependent credibility and no combined effects is shown in the top right panel. All QTLs are detected. Including the combined effects in the analysis would result in a large number of yellow and light blue peaks just as in the ordinary PW map in the middle left panel. However, such inference is more sensitive and it detects locus 7 (see S1 Fig.).

**Fig 8:**
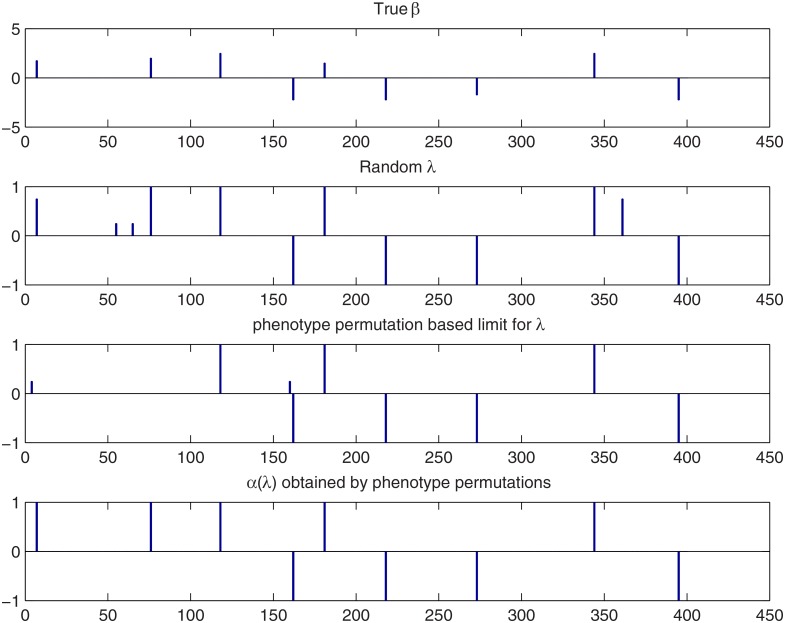
Comparison of the different detection methods with the Bayesian LASSO for the simulated Wheat data when combined effects are not considered. Top panel: the true QTL effect vector *β*. Second panel: the results for HPW inference with a random *λ*. Third panel: the results for HPW inference by limiting *λ* using phenotype permutation. Bottom panel: the results for *λ*-dependent credibility threshold. See Section 3.1.2 for the interpretation of the bar heights.

Fig. 8 summarizes the results for the different approaches when combined effects are included in the analyses. We note that this time treating *λ* as random in HPW inference produces the falsely positive sum *β*_65_+*β*_361_ with the non-existent effect *β*_361_ flagged as the stronger partner.

### 3.3 Barley data

**Fig 9:**
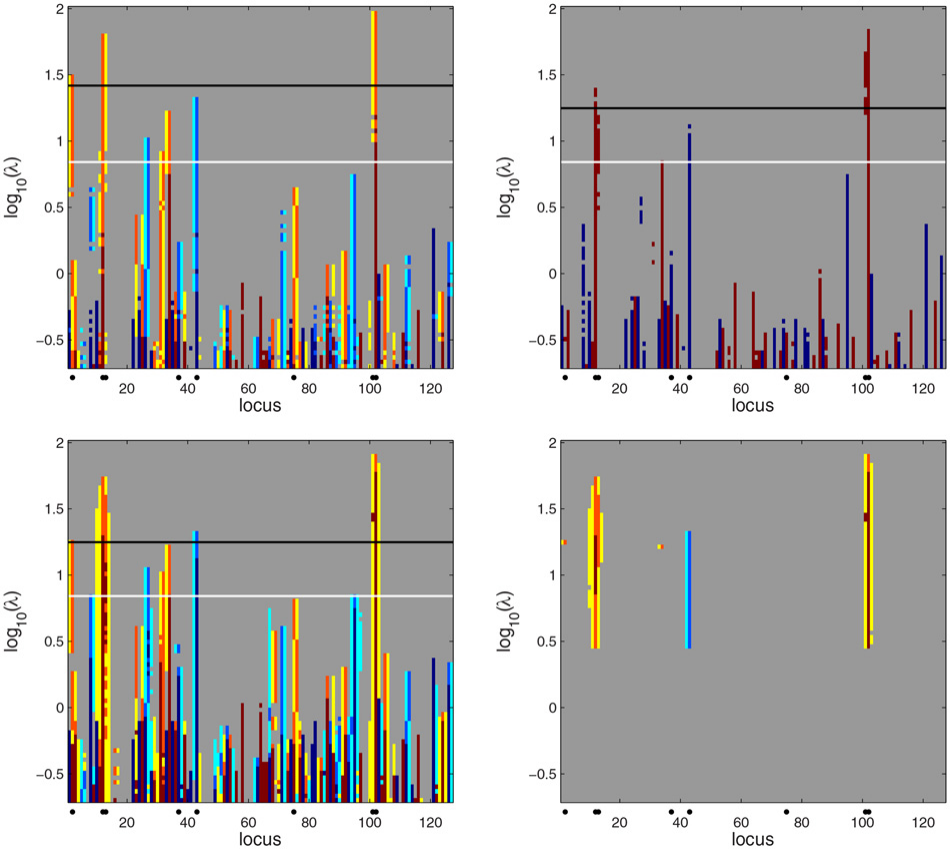
Scale space Bayesian LASSO QTL analysis for the real Barley data set. Upper left and right: HPW credibility maps with and without combined effects. Lower left and right: PW credibility maps with combined effects using either a fixed or a *λ*-dependent credibility threshold. The white horizontal line marks the posterior mean of a random *λ*. The black line marks the phenotype permutation based threshold for *λ*. For color interpretation see captions of Fig. 1 and Fig. 3.

In our last example we analyze the real Barley data set described in [40] using the average value of kernel weight over environments as the observed phenotype. This time the missing values in **X** were not imputed but instead set to the neutral value 0. The posterior sampling scheme and the scale space *λ*-range were the same as with the simulated Barley example in Section 3.1. The HPW QTL credibility maps with and without combined effects are shown in the upper panels of Fig. 9. The PW maps with combined effects using either a fixed or a *λ*-dependent credibility level are shown in the bottom panels. As before, the threshold for *λ* obtained with phenotype permutation is marked with a black line and the posterior mean of a random *λ* is marked with a white line.

Since we now deal with a real data set, the true QTLs are not known. However, to compare our methods with other approaches, we have denoted by black dots in the lower parts of the maps the loci detected using LASSO, an elastic net or adaptive LASSO in [43] as well as the ones detected in [44] using an empirical Bayes method (E-BAYES) that aims to map epistatic QTLs under the mixed model framework. In E-BAYES, six most important markers are detected without any specific decision making process. In LASSO, elastic net and adaptive LASSO the multi-split method of Meinshausen et al. [45] was used. In that case, different combinations of markers 2, 11 or 12 and 101 or 102 were detected. In addition to these, markers 37, 43 and 75 were detected with E-BAYES.

**Fig 10:**
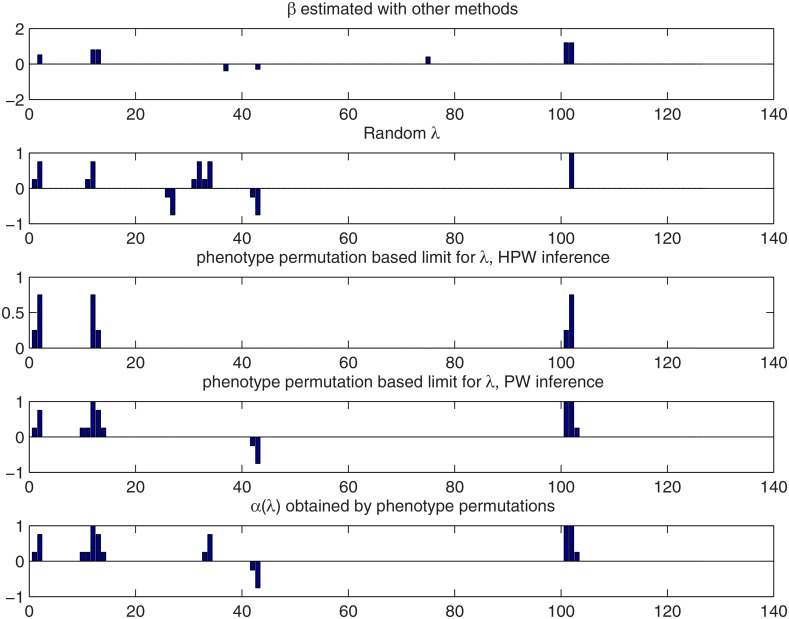
Comparison of the different detection methods with the Bayesian LASSO for the real Barley data set when combined effects are considered. Top panel: the estimated effect vector *β*. Second panel: the results for HPW inference with a random *λ*. Third and fourth panels: the results for HPW and PW inference by limiting *λ* using phenotype permutation. Bottom panel: the results for *λ*-dependent credibility threshold. See Section 3.1.2 for the interpretation of the bar heights.

In the HPW and PW maps with the combined effects (top and bottom left in Fig. 9), there are several peaks that stick out of an apparent background of false positives. However, the phenotype permutation based limit of *λ* (black line) is rather conservative with HPW inference, flagging only the loci 2, 12 and 102 as QTLs. Using PW inference with a *λ*-dependent credibility level (lower right panel of Fig. 9) also the markers 34 and 43 are detected while PW inference detects loci 2, 12, 102 and 43 and almost locus 34 (left lower panel of Fig. 9). Random *λ* based limit (white line) is again the most liberal adding the loci 27 and 32 to the set of QTLs. The different detection methods are compared using bar plots in Fig. 10.

The replacement of missing values with zero may cause combinations where the percentage of *x*_*lj*_ ≠ *x*_*lk*_ exceeds *ρ* due to the zero values. In this dataset there were 16 such combinations. However, none of the corresponding effects could be considered credible at a reasonable level of *λ*.

If combined effects are not included in the analysis, the peak at locus 2 does not exceed the permutation based limit in HPW inference (upper right panel of Fig. 9) and with a random *λ* the loci 2 and 27 and 32 are not detected in HPW inference (see panel 3 of S2 Fig.). Further, in PW inference with either a fixed or a *λ*-dependent credibility level only the loci 12, 43 and 102 are detected (see S3 Fig.).

## Discussion

The tuning parameter *λ* plays an important role in Bayesian LASSO regression and it is usually treated as a random variable. In the spirit of scale space methodology, we proposed to consider instead a whole range of *λ*’s in the analyses and visualize the results with maps that sum up inferences and help identify the important explanatory variables. In this way information about the sensitivity of the results to the tuning parameter value is obtained and one does not have to blindly trust the results obtained with a random *λ*. Compared to the visualization tools provided by other approaches that utilize a range of *λ*’s, the advantage of our scale space maps is the possibility to locate the QTL candidates with one glance.

As the posterior mean of noise variance depends on the tuning parameter, a large number of false positives are created when *λ* is too small. Visual inspection can then be used to associate the highest peaks in a scale space map with important explanatory variables. We also proposed a permutation based lower bound for useful values of the tuning parameter. As a completely different approach, we let the credibility level in marginal posterior inference depend on the value of *λ*. Other important goals were handling of collinearity among the explanatory variables and use of simultaneous inference to correct for multiplicity in hypothesis testing. Collinearity was taken into account by including combined effects, that is, sums and differences of regression coefficients in the inference. The method of highest point-wise probabilities was used for simultaneous inference (cf. [17]). When Bayesian shrinkage models are applied to genetic datasets, the decision making to judge QTLs is often based on frequentist reasoning and phenotype permutation is typically used to correct for multiple testing [8, 9]. In contrast, the HPW method used here is a Bayesian procedure and we therefore deviated from a fully Bayesian paradigm only to control for the false positives with small values of *λ*.

The novel methods were tested on three genetic data sets where the goal was to associate genetic markers with observed phenotypes. The design matrix in all of them was taken from a real data set but in the first two examples the observed phenotypes were artificially constructed. In the third example, all data was real. In the first artificial data set, neighboring explanatory variables were collinear and we mimicked a situation where the true QTL is located between two markers by removing some QTL loci before the analysis. If collinearity was not taken into account, some QTLs were not detected and considerably better results were obtained by including combined effects in the analysis. For the real data set, fewer loci were flagged as QTLs when the combined effects were not considered. In the second artificial example, none of the QTLs were removed before the analyses and all QTLs were detected without including combined effects.

The phenotype permutation based limit for a *λ*-dependent credibility level worked well for all three data sets. On the other hand, the permutation based limit for *λ* seems to be somewhat more conservative, especially when simultaneous inference was applied. Still, we feel that the latter approach offers more insight into the data. As both maps can be drawn at the computational cost of one, we recommended to explore both. In all three examples, more credible variables were detected with a random *λ* than by either permutation based approach. However, a random *λ* may lead to too liberal detection, as it produced one false positive for both simulated data sets.

In on-line supplement S1 Appendix we also demonstrated the use of scale space analysis for Bayesian ridge regression and the classic LASSO. Unlike LASSO, ridge regression has been criticized for not being able to shrink any of the regression coefficients to zero and therefore being incapable of proper variable selection. Moreover, according to the empirical results of Park and Casella [3], Bayesian LASSO estimates of the regression coefficients tended to be somewhere between the estimates produced by LASSO and ridge regression. This is consistent with our findings. The truly non-zero coefficients were easy to identify from the map of LASSO estimates while in the posterior mean maps for Bayesian ridge regression and the Bayesian LASSO, the peaks at the QTLs were much harder to discern. LASSO also tends to select only one variable from a set of collinear variables [46] and therefore does not seem to suffer from collinearity as much as its Bayesian version or Bayesian ridge regression. However, using our technique with the combined effects included, non-zero coefficients were detected with both Bayesian LASSO and Bayesian ridge regression that produced comparable results in this setting.

A scale space version of the classic LASSO combined with a permutation test led to a rather conservative detection strategy and consequently many QTLs were missed. However, even fewer QTLs might be detected if, instead of the scale space view, the value of the LASSO tuning parameter *λ* were estimated from the data using for example BIC or cross-validation. Therefore, even with the classic LASSO, the scale space approach is valuable in that it provides a means to evaluate the effect of the tuning parameter on the inference at a glance.

The number of loci included in the model has been moderate in our examples. Much larger datasets could be analyzed by first screening for the most effective loci using for example the sure importance screening suggested in [47] and applying then the Bayesian LASSO to the resulting smaller subset of loci.

The Matlab codes used for computations together with instructions for its use and examples are available at http://cc.oulu.fi/~lpasanen/SSBLASSO. The codes are also available at lib.stat.cmu.edu/general/SSBLASSO.zip. Posterior sampling was carried out using the modified BLR-function from R-package BLR [41] but all the computations can be done in Matlab because a tailor-made interface is provided for running the R scripts from within Matlab. Modification of the BLR-function so that posterior samples of the coefficients are saved facilitates also for example the computation of the posterior distribution of the function of the marker effects including heritability and genomic breeding values.

When computing the Bayesian LASSO scale space maps, MCMC posterior sampling is the most time consuming part. As an example, for the artificial Wheat data, using a Dell OptiPlex 9010 PC with Intel Core i7-3770 CPU and 32Gb of RAM under Windows 7 64-bit operating system, one MCMC run takes 90 seconds while the subsequent posterior inference takes only 4 seconds. For a scale space map with *r* *λ*-values and *p* phenotype permutations, *pr*+*r* distinct MCMC chains need to be run. However, as all these sampling steps are independent of each other, computations can be parallelized either by using several computers or multiple processors.

Another possibility for a speed-up might be to use importance sampling [48]. Since, for a given phenotype vector, the posterior sample changes only little between subsequent values of *λ*, one could transform the MCMC sample obtained with *λ*_*i*_ to a sample that corresponds to *λ*_*i*+1_. With this approach one could compute a scale space credibility map with just *p*+1 MCMC chains. Such an idea has been used with profile of likelihood ratio statistic in [49]. Sequential Monte Carlo implementation for a similar problem was presented in [29].

A tempting approach to phenotype permutation would also be the “permutation within Markov chain” technique proposed by Che and Xu [50], where the permutations are carried out within a single chain. With this approach only 2*r* MCMC samples would be needed for a scale space credibility map. Further, if this idea is combined with importance sampling, only two MCMC chains would be needed. However, no theoretical justification has yet been provided for the permutation withing Markov chain procedure.

A simple method to speed up the search for the *λ*-limit would also be to first consider only few phenotype permutations with a substantially sparser *λ*-grid than the one used in the scale space map. This small phenotype permutation sample can be used to identify a *λ*-interval that contains the desired limiting value. Finally, a larger number of permutations are generated for this short interval with the same *λ*-value density employed in the scale space maps.

Finally we want to point out that while the focus of this paper has been on genetic datasets with dichotomic explanatory variables, the proposed methods are likely to be applicable also in other contexts and with other types of explanatory variables. The scale space approach could also be used to select hyperparameters for other common prior distributions used in Bayesian variable selection (e.g. [1, 51]).

## Supporting Information

S1 Appendix Bayesian ridge regression and classic LASSO. **Figure A in S1 Appendix: Scale space view of Bayesian ridge regression for the simulated Barley data.** Top left: quantized estimated posterior mean effects for a range of tuning parameters 684 λ see caption of Fig. 1 for interpretation of colors. Top right: credibility map using a λ-dependent credibility threshold. Bottom panels: PW and HPW credibility maps. In all credibility maps combined effects are included in the analysis. For color coding see the caption of Fig. 3. The white horizontal line marks the posterior mean of a random λ. The Black line marks the phenotype permutation based threshold for λ.
**Figure B in S1 Appendix: Scale space view of LASSO for the simulated Barley data.** Left panel: estimates of the posterior mean effects for a range of tuning parameters λ. Right panel: scale space inference based on a permutation test. Red and blue flag the significantly positive and negative effects. The estimates of λ obtained with BIC and cross-validation are marked with yellow and green lines, respectively.
**Figure C in S1 Appendix: Scale space view of Bayesian ridge regression for the real Barley data.** Left: quantized estimated posterior mean effects for a range of tuning parameters λ see caption of Fig. 1 for interpretation of colors. Right: HPW credibility map with combined effects combined effects. For color coding see the caption of Fig. 3. The white horizontal line marks the posterior mean of a random λ. The Black line marks the phenotype permutation based threshold for λ.
**Figure D in S1 Appendix: Scale space view of LASSO for the real Barley data.** Left panel: estimates of the posterior mean effects for a range of tuning parameters λ. Right panel: scale space inference based on a permutation test. Red and blue flag the significantly positive and negative effects. The estimates of λ obtained with BIC and cross-validation are marked with yellow and green lines, respectively.
(PDF)

S1 Fig Scale space Bayesian LASSO QTL analysis of the simulated Wheat data. The PW QTL credibility analyses with the combined effects with a *λ*-dependent credibility threshold.
(EPS)

S2 Fig QTL credibility analysis of real Barley data with the Bayesian LASSO using a random **λ**. Top panel: The estimates of *β* obtained with other methods. Second and third panels: the loci with credibly non-zero effects detected using point-wise and simultaneous inference, respectively. Here 1 corresponds to a credibly positive locus and -1 corresponds to a credibly negative locus. Fourth and fifth panels: the loci with credibly non-zero coefficients detected using point-wise and simultaneous inference when also combined effects are considered. See the text for the interpretation of the bar heights.
(EPS)

S3 Fig QTL credibility analysis for the real Barley data with PW inference without the combined effects. Left: Fixed credibility level. Right: *α*-dependent credibility level.
(EPS)
