# Supplementary material for: Bayesian LASSO, Scale Space and Decision Making in Association Genetics
Source: PLoS One. 2015 Apr 9;10(4):e0120017. doi: 10.1371/journal.pone.0120017 (PMC4391919; doi:10.1371/journal.pone.0120017)
Supplement: S1 Appendix — Figure A in S1 Appendix: Scale space view of Bayesian ridge regression for the simulated Barley data. Top left: quantized estimated posterior mean effects for a range of tuning parameters 684 λ see caption of Fig. 1 for interpretation of colors. Top right: credibility map using a λ-dependent credibility threshold. Bottom panels: PW and HPW credibility maps. In all credibility maps combined effects are included in the analysis. For color coding see the caption of Fig. 3. The white horizontal line marks the posterior mean of a random λ. The Black line marks the phenotype permutation based threshold for λ. Figure B in S1 Appendix: Scale space view of LASSO for the simulated Barley data. Left panel: estimates of the posterior mean effects for a range of tuning parameters λ. Right panel: scale space inference based on a permutation test. Red and blue flag the significantly positive and negative effects. The estimates of λ obtained with BIC and cross-validation are marked with yellow and green lines, respectively. Figure C in S1 Appendix: Scale space view of Bayesian ridge regression for the real Barley data. Left: quantized estimated posterior mean effects for a range of tuning parameters λ see caption of Fig. 1 for interpretation of colors. Right: HPW credibility map with combined effects combined effects. For color coding see the caption of Fig. 3. The white horizontal line marks the posterior mean of a random λ. The Black line marks the phenotype permutation based threshold for λ. Figure D in S1 Appendix: Scale space view of LASSO for the real Barley data. Left panel: estimates of the posterior mean effects for a range of tuning parameters λ. Right panel: scale space inference based on a permutation test. Red and blue flag the significantly positive and negative effects. The estimates of λ obtained with BIC and cross-validation are marked with yellow and green lines, respectively. (PDF) [file pone.0120017.s001.pdf]

Supporting Information for 'Bayesian LASSO, scale space and decision making in association genetics'

Leena Pasanen[1*], Lasse Holmström[1] and Mikko J. Sillanpää[1,2,3]

1 Department of Mathematical Sciences, University of Oulu, Finland
2 Department of Biology, University of Oulu, Finland
3 Biocenter Oulu, Finland
∗ E-mail: Corresponding Leena.Pasanen@oulu.fi

1 Bayesian ridge regression and classic LASSO

1.1 Bayesian ridge regression

In ridge regression [4], the estimate of $\boldsymbol{\beta}$ is obtained using L_2-constrained least squares,

$$\widehat{\boldsymbol{\beta}} = \arg\min_{\boldsymbol{\beta}} \left(\|\mathbf{y} - \mu\mathbf{1} - \mathbf{X}\boldsymbol{\beta}\|^2 + \lambda\|\boldsymbol{\beta}\|^2 \right). \tag{S1}$$

The larger the value of λ, the more the components of $\boldsymbol{\beta}$ are shrunk towards zero.

A Bayesian view of ridge regression is obtained by noting that the minimizer of (S1) can be considered as the posterior mean of a model where $\beta_j \sim \mathrm{N}(0, \sigma^2/\lambda)$, for all j [4] (see also [5]). However, Bayesian ridge regression is used relatively rarely in practice. In our experiments with Bayesian ridge regression we followed [2] and used the model (1) with an unscaled Gaussian prior for the regression coefficients, $\beta_j \sim \mathrm{N}(0, 1/\lambda)$, for all j.

We tried the ideas described in the previous sections also with Bayesian ridge regression. Again, the simulated Barley data set was used in the tests. The MCMC simulation was set up exactly as with the Bayesian LASSO (Section 3.1) and the BLR R-library again was used. For scale space analysis, the λ-range included 80 values from $10^{-0.7}$ to 10^2 with a logarithmic spacing.

The quantized posterior effect means are displayed in the top left panel of Figure A. The credibility map based on the function $\alpha(\lambda)$ is in the top right panel and the QTLs detected using PW and HPW inference are shown in the bottom panels. The phenotype permutation based limit (obtained using 100 permutations) and the posterior mean of a random λ are marked with black and white lines, respectively. Ridge regression has been criticized for shrinking all regression coefficients equally and therefore not being able to realize proper variable selection. The effects of collinear loci in particular are thought remain strongly inflated. However, judging from Figure A, this problem can be largely fixed by including combined effects in the analysis because, a bit surprisingly, the results are rather similar with those obtained with the Bayesian LASSO (Figures 3 and 5). All QTL effects can be easily visually separated from false positives, both with PW and HPW inference.

Some differences between the Bayesian LASSO and Bayesian ridge regression are also apparent. First, inference based on a λ-dependent credibility threshold $\alpha(\lambda)$ is more liberal and false positives appear for small values of λ. Second, the phenotype permutation based limit for λ is much lower for ridge regression. This may be explained by the different shrinkage priors used by the Bayesian LASSO and Bayesian ridge regression. The Gaussian prior in Bayesian ridge regression shrinks all effects more heavily than the Laplacian prior of the Bayesian LASSO so that under the null hypothesis, the λ-value above which no false positives appear is smaller. One consequence is that detection of the highly collinear locus (48,49) is much easier with Bayesian ridge regression.

1.2 Classic LASSO

The scale space approach can also be applied with classic, non-Bayesian LASSO regression. The estimation results for the simulated Barley data are shown in the left panel of Figure B. The estimates for λ obtained using BIC and 5-fold cross-validation (see e.g. [3]) are marked by yellow and green lines, respectively. In the left panel,

the QTLs seem to produce more clearly defined peaks than was the case with the Bayesian LASSO or Bayesian ridge regression. Therefore, there was no need to quantize the colors. The result is consistent with the view that LASSO performs variable selection more efficiently than its Bayesian version or Bayesian ridge regression. LASSO is also known to tend to select only one variable from a set of collinear variables [8] and therefore the effects are not heavily spread over the neighboring loci. This phenomenon is clearly visible here as the effect of the strongly collinear loci (48,49) has not spread over to the neighboring loci, and its color is as bright as at the other QTLs. Thus, LASSO does not seem to suffer from collinearity as much as its Bayesian counterpart or Bayesian ridge regression.

The right panel of Figure B shows scale space credibility analysis for LASSO when a permutation test based 95% quantile was used as the cut-off. As a simultaneous inference method, permutation test is conservative and some QTLs are missed. However, even fewer significant regression coefficients would be detected if, instead of scale space analysis, only BIC or CV estimates would be employed for λ (see the yellow and green lines in the right panel of Figure B). For point-wise inference, the multi-split method of Meinshausen et al. [7] or confidence intervals with bootstrapping [1] could be used. Even without including combined effects and with the conservative permutation test, LASSO is able to detect the loci (48,49) which the Bayesian LASSO had difficulties to find. Again, this is in line with the assumption that LASSO does not suffer from collinearity as much as its Bayesian version or Bayesian ridge regression (cf. [6]).

1.3 Barley data

Scale space versions of Bayesian ridge regression and the classic LASSO were also tested for the Barley data, including combined effects in the analysis. The results of ridge regression are shown in Figure C. For ridge regression, the loci 1, 12, 32, 34, 43 and 102 and some of their neighbors exceeded the permutation test based limit in HPW inference. As shown in Figure D, with LASSO, the markers 2, 12, and 102 were detected with $\lambda = 10^{-0.46}$ when a permutation test was used. However, if BIC was used to pick λ, only the strongest markers 12 and 102 were detected with the permutation test and with CV no detections were made. Therefore, the combination of a scale space and a permutation test seems to be helpful also with classic LASSO.

References

[1] Arindam Chatterjee and Soumendra Nath Lahiri. Bootstrapping Lasso estimators. *Journal of the American Statistical Association*, 106(494):608–625, 2011.

[2] Gustavo de los Campos and Paulino Perez Rodriguez. *BLR: Bayesian Linear Regression*, 2012. R package version 1.3.

[3] Trevor Hastie, Robert Tibshirani, and Jerome Friedman. *The elements of statistical learning: Data mining, inference and prediction*. Springer, New York, 2 edition, 2009.

[4] Arthur E Hoerl and Robert W Kennard. Ridge regression: Biased estimation for nonorthogonal problems. *Technometrics*, 12(1):55–67, 1970.

[5] T. C. Hsiang. A Bayesian view on ridge regression. *Journal of the Royal Statistical Society: Series D*, 24(4):267–268, 1975.

[6] Zitong Li and Mikko J. Sillanpää. Overview of LASSO-related penalized regression methods for quantitative trait mapping and genomic selection. *Theoretical and Applied Genetics*, 125(3):419–435, 2012.

[7] Nicolai Meinshausen, Lukas Meier, and Peter Bühlmann. P-values for high-dimensional regression. *Journal of the American Statistical Association*, 104(488):1671–1681, 2009.

[8] Hui Zou and Trevor Hastie. Regularization and variable selection via the elastic net. *Journal of the Royal Statistical Society: Series B*, 67(2):301–320, 2005.

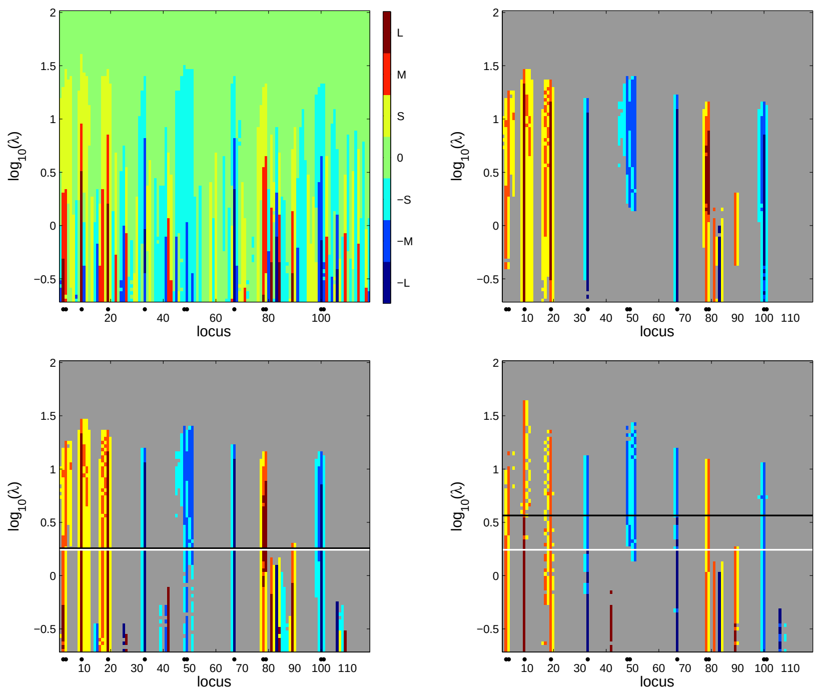

Figure A: **Scale space view of Bayesian ridge regression for the simulated Barley data.** Top left: quantized estimated posterior mean effects for a range of tuning parameters λ; see caption of Figure 1 for interpretation of colors. Top right: credibility map using a λ-dependent credibility threshold. Bottom panels: PW and HPW credibility maps. In all credibility maps combined effects are included in the analysis. For color coding see the caption of Figure 3. The white horizontal line marks the posterior mean of a random λ. The Black line marks the phenotype permutation based threshold for λ.

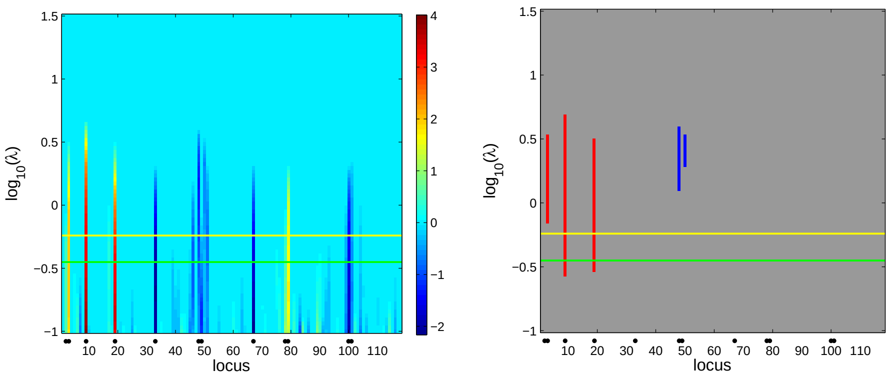

Figure B: **Scale space view of LASSO for the simulated Barley data.** Left panel: estimates of the posterior mean effects for a range of tuning parameters λ. Right panel: scale space inference based on a permutation test. Red and blue flag the significantly positive and negative effects. The estimates of λ obtained with BIC and cross-validation are marked with yellow and green lines, respectively.

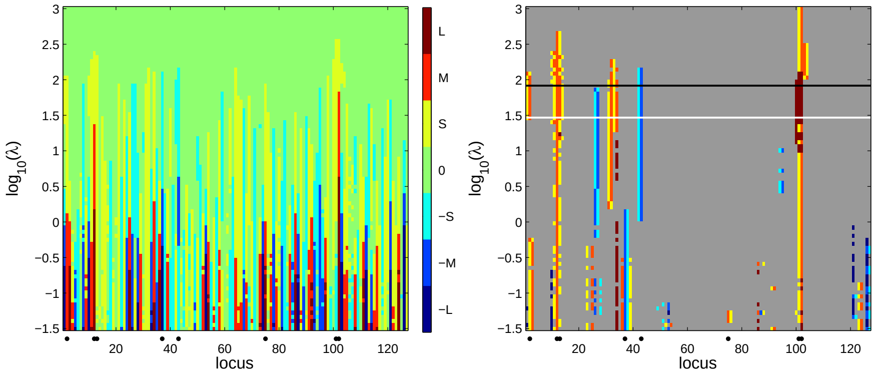

Figure C: **Scale space view of Bayesian ridge regression for the real Barley data.** Left: quantized estimated posterior mean effects for a range of tuning parameters λ; see caption of Figure 1 for interpretation of colors. Right: HPW credibility map with combined effects combined effects. For color coding see the caption of Figure 3. The white horizontal line marks the posterior mean of a random λ. The Black line marks the phenotype permutation based threshold for λ.

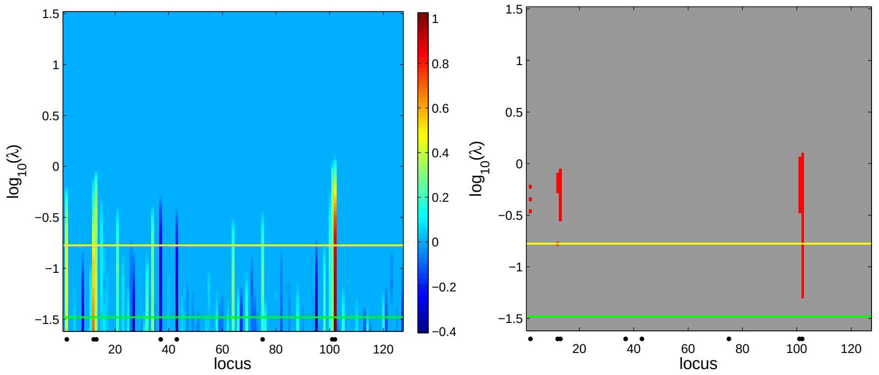

Figure D: **Scale space view of LASSO for the real Barley data.** Left panel: estimates of the posterior mean effects for a range of tuning parameters λ. Right panel: scale space inference based on a permutation test. Red and blue flag the significantly positive and negative effects. The estimates of λ obtained with BIC and cross-validation are marked with yellow and green lines, respectively.
